# Cardiac screening in the young for the detection of cardiovascular disease and prevention of sudden cardiac death

**DOI:** 10.1371/journal.pmed.1005278

**Published:** 2026-10-22

**Authors:** Daniel T. Tardo, Sanjay Sharma

**Affiliations:** 1 Cardiovascular and Genomics Research Institute, City St. George’s, University of London, London, United Kingdom; 2 St. George’s University Hospitals NHS Foundation Trust, London, United Kingdom; 3 School of Medicine, The University of Notre Dame Australia, Sydney, Australia; 4 Heart, Vascular and Thoracic Institute, Cleveland Clinic London, London, United Kingdom

## Abstract

In this Perspective, Daniel Tardo and Sanjay Sharma discuss evidence that supports cardiac screening in the young as a preventative strategy for cardiovascular disease and sudden cardiac death. They highlight the need for structured screening frameworks, and discuss the potential of AI-enabled technologies for disease detection and risk prediction.

## Teaser

Sudden cardiac death in the young is often due to silent inherited cardiac conditions. Although evidence increasingly supports cardiac screening as a preventative strategy, policies and frameworks differ globally. Structured screening frameworks that utilise AI-enabled technologies show great potential for enhancing disease detection and risk prediction.

## Introduction

Cardiovascular disease (CVD) is a major cause of death in individuals aged under 35, with an estimated global mortality rate of 10–25 deaths per 100,000 person-years. In this age group, sudden cardiac arrest (SCA) and sudden cardiac death (SCD) are most often caused by rare inherited and congenital cardiac conditions, which are frequently clinically silent [1]. Most existing data derive from athletic cohorts, with supporting insights from non-athletic cohorts. In the UK, rates of SCD in adolescent football players occur at an incidence of 1 in 14,794 person-years [2], whereas the Office for National Statistics data estimate 1 in 55,000 person-years in the young general population [3]. Recent data from our group suggest the latter is a significant underestimate, with an SCD incidence of 1 in 31,250 person-years, rising to 1 in 17,901 when survived SCA is included [1]. In Denmark, a 20-year nationwide study of individuals aged 1–35 years reported an overall SCD incidence of 2.2 per 100,000 person-years (approximately 1 in 45,500), with a 49% decline between 2000 and 2019 [4]. This decline coincided with a 10-fold increase in the diagnosis of inherited cardiac conditions, a doubling in implantation rates of implantable cardioverter-defibrillators (ICDs), and improved survival following out-of-hospital cardiac arrest, largely driven by increased bystander cardiopulmonary resuscitation and defibrillation [4]. Despite this, SCA/SCD in the young remain a significant public health issue, and effective primary prevention depends on early disease detection through proactive testing.

Cardiac screening combines a focussed health questionnaire pertaining to cardiac symptoms and family history of premature CVD/death, physical examination, 12-lead electrocardiogram (ECG) and, where resources allow, echocardiography. International practice varies primarily in the use of the ECG. Being the diagnostic test of choice for electrical disorders and capable of raising suspicion of structural disease, including cardiomyopathies, the ECG allows a proportion of inherited conditions predisposing to SCA/SCD to be identified. While targeted screening of higher-risk individuals is widely implemented in the UK, population-based screening of asymptomatic individuals remains controversial.

## Cardiac screening recommendations and international experience

The European Society of Cardiology and American Heart Association support pre-participation screening in young athletes, and many sporting organisations mandate screening of elite athletes. In contrast, the UK National Screening Committee concluded in 2019 that a national cardiac screening programme did not meet criteria, citing uncertainty regarding SCD incidence, the absence of an accurate and validated screening test, and limited evidence supporting treatment of inherited cardiac conditions. Since then, incidence and diagnostic yield have been further characterised, and international guidelines now provide evidence-based treatment strategies [5,6].

The evidence addressing whether screening reduces SCD is predominantly observational, in the absence of randomised trials. Italy has mandated screening of competitive athletes by federal law since 1982; SCD incidence among screened athletes fell by almost 90% over 25 years, while remaining unchanged in unscreened non-athletes [7]. This was attributed to the diagnosis of cardiomyopathies and subsequent disqualification from competitive sport. This concurrent comparator group is the study’s principal strength, although the data derive from the Veneto region alone (9% of the national population); arrhythmogenic right ventricular cardiomyopathy was being characterised as the main cause of exercise-related SCD over the same interval, and the elevated baseline incidence raises the possibility of regression to the mean. Improving disease recognition and expanding defibrillator access therefore cannot be fully disentangled from the intervention of cardiac screening.

Israel introduced comparable legislation in 1997 but found SCD incidence essentially unchanged (2.54 vs. 2.66 per 100,000 person-years) [8]. This null result argues against a large effect, though newspaper-based case ascertainment in the Israeli study likely underestimated event numbers, and a pre-programme cluster of cases means the lower subsequent rates may equally reflect regression to the mean.

Japan has screened all schoolchildren in the first, seventh and 10th grades since 1973, irrespective of sport participation [9]. This general population programme supports the sensitivity of the ECG, with 2.7% of those screened requiring secondary evaluation and a prevalence of 0.024% for SCD-associated conditions.

In the UK, the charity Cardiac Risk in the Young (CRY) has screened individuals aged 14–35 on a voluntary basis for over 30 years, generating a heterogeneous cohort reflective of the general population that also incorporates athletes (9%). The programme offers a mobile screening service throughout the UK, comprising a health questionnaire, ECG interpreted using international guidelines, selective echocardiography, and cardiology consultation (Fig 1) [10]. A recent analysis of 104,369 individuals screened between 2008 and 2018, with outcomes ascertained through national datasets, participant survey, and direct reporting, found that among the 2.5% (n = 2,619) referred after initial evaluation, 280 (0.27%) were diagnosed with an SCA/SCD-associated condition, of whom 41% received risk-modifying interventions [1]. Sensitivity for detecting these conditions was 32.5% and 75.4% for the questionnaire and ECG, respectively. Conversely, during a follow-up period of 6 years, 0.08% of the cleared individuals were diagnosed with an SCA/SCD-associated condition.

**Fig 1:**
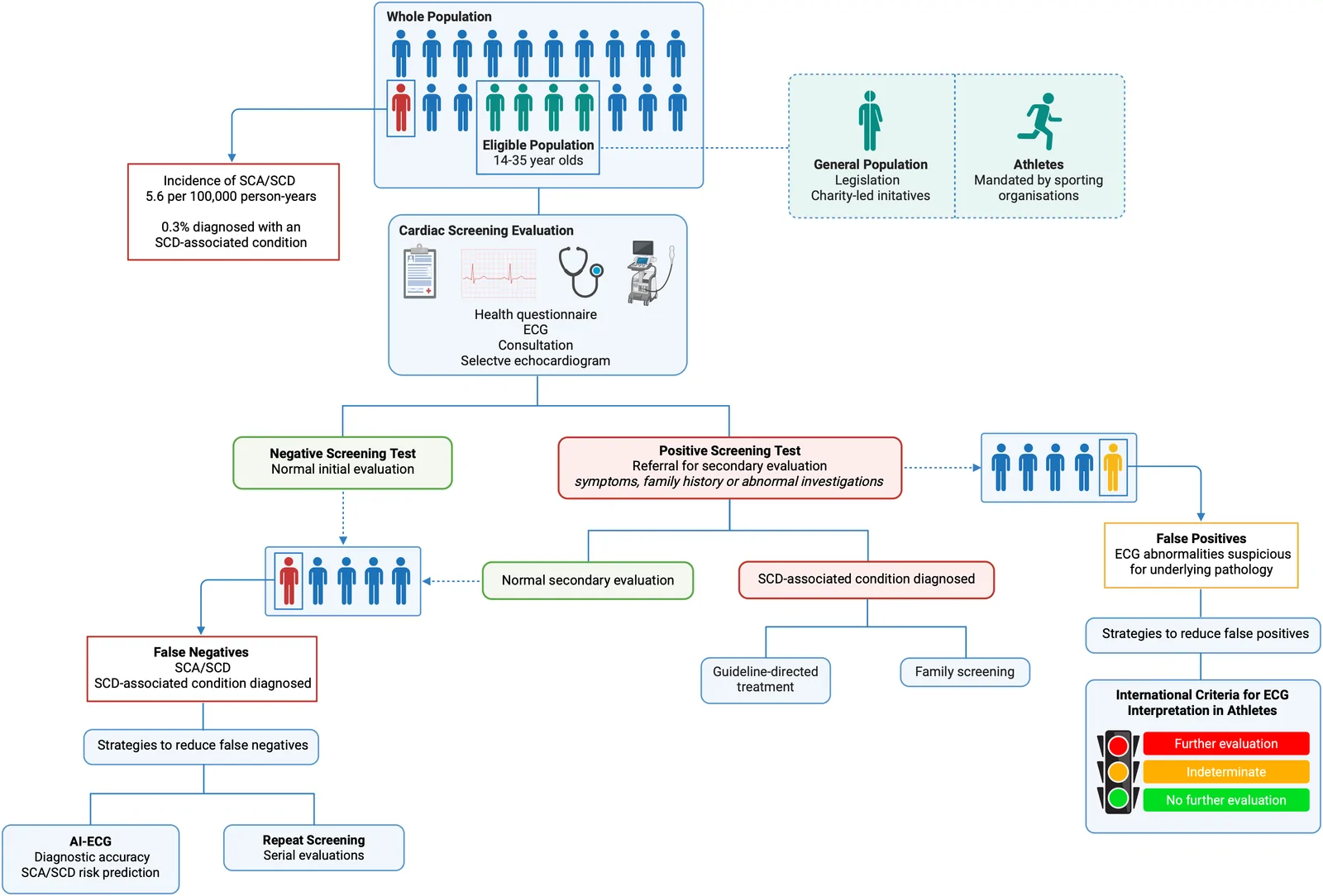
A framework for cardiac screening in the young. Cardiac screening in the young aims to identify individuals at risk of sudden cardiac arrest (SCA) and sudden cardiac death (SCD) by diagnosing rare inherited cardiac conditions. Screening programme design varies by target population, namely the general population vs. elite athletes, and correspondingly follows relevant government legislation or mandated protocols. Evaluation typically combines a health questionnaire, physical examination, electrocardiogram (ECG), echocardiogram, and consultation, with the specific combination varying by location, clinical context and sport. Applying the international consensus statement for ECG interpretation has been shown to reduce false-positives. Strategies to reduce false-negatives include repeat screening (to account for age-related penetrance) and implementation of artificial intelligence-enabled electrocardiogram (AI-ECG), which has the potential to enhance diagnostic accuracy and SCA/SCD risk prediction; however, validation in young cohorts is still required. Created in BioRender. Tardo, D. (2026) https://BioRender.com/o0yn3k7.

Collectively, these findings support an important role for cardiac screening in preventing SCD in both young athletic and non-athletic populations. Nonetheless, consideration must be given to the challenges influencing how such programmes are delivered.

## Challenges and clinical implications of cardiac screening

ECG interpretation depends on expertise and is prone to false positives, with significant inter-observer variability. The International Criteria for ECG Interpretation in Athletes help distinguish physiological adaptation from underlying cardiovascular pathology, and have improved both specificity and sensitivity relative to earlier iterations [10]. Their applicability to general population screening, namely implementation of the ‘abnormal ECG findings’ category used to identify changes suggestive of pathology, has been demonstrated by CRY’s programme [1].

False negatives occur in concealed channelopathies and cardiomyopathies, and some causes of SCD, including sudden arrhythmic death syndrome, coronary anomalies and idiopathic left ventricular hypertrophy, may only be diagnosed at post-mortem examination. Among the 97.5% (n = 101,750) cleared at initial evaluation in the CRY cohort, 86 individuals (0.08%) were subsequently diagnosed with an SCA/SCD-associated condition at a mean of 4 years, including 29 cases of SCA/SCD [1]. CRY offers a single evaluation, raising the issue of age-related penetrance and repeat screening. In an Italian cohort of 22,300 children followed over 11 years, repeat evaluation increased diagnostic yield by 60% [11].

## Could artificial intelligence address these challenges?

The above limitations underscore the need to optimise ECG interpretation, which the Lancet Commission on SCD acknowledged may be achieved by artificial intelligence (AI) [12]. AI-enabled ECG (AI-ECG) applies machine learning and deep neural networks to interpret large volumes of ECGs, autonomously identifying complex diagnostic features and offering the potential for personalised risk assessment [13]. In older populations, AI-ECG permits risk stratification for overall and cardiovascular mortality, heart failure and arrhythmias [13]. Interestingly, one study applied deep learning to a population-wide Swedish cohort of ECGs linked to death certificates, identifying a high-risk group with a 7% annual SCD rate, exceeding that of individuals with reduced left ventricular ejection fraction (LVEF) considered eligible for ICDs. Importantly, 86.1% of the high-risk cases would not have been identified by LVEF [14]. This model also revealed a previously undescribed ECG biomarker, illustrating the potential of AI-ECG.

Applying such models to the young poses distinct challenges. Firstly, the rarity of SCA/SCD events makes these outcomes difficult to predict, limiting the positive predictive value. Secondly, the underlying disease substrate is inherited rather than acquired, which differs from the majority of large-scale AI-ECG studies conducted to date. Furthermore, ECG features unique to the athlete’s heart must be interpreted in the correct context, to avoid being mistaken as false positive findings by an AI-ECG model. Similarly, this is the case for calibrating models to detect differences based on sex, gender and ethnicity. These limitations highlight the need to incorporate sufficient training data to ensure future models are specific to athletic and non-athletic young individuals. Sex and gender differences, the impact of ethnicity, cost-effectiveness, and access to and uptake of screening also remain underexplored.

## Conclusions

Cardiac screening in the young increasingly satisfies established criteria for a justified programme. It addresses an important health problem with a latent phase, utilises an inexpensive and acceptable test whose accuracy has improved with the International Criteria and has the potential to improve with AI-ECG; in support, evidence-based treatments for the conditions detected are well-established. The outstanding questions concern how such a programme is best delivered alongside emergency action planning, rather than whether it should exist. Sustained engagement with funders and policymakers is required so that preventing SCD in the young becomes a matter of public health policy, not solely a charitable endeavour.

## Abbreviations

AI: artificial intelligence
AI-ECG: AI-enabled ECG
CRY: Cardiac Risk in the Young
CVD: Cardiovascular disease
ECG: electrocardiogram
ICDs: implantable cardioverter-defibrillators
LVEF: left ventricular ejection fraction
SCA: sudden cardiac arrest
SCD: sudden cardiac death
